# Using Content Analysis to Characterise the Sensory Typicity and Quality Judgements of Australian Cabernet Sauvignon Wines

**DOI:** 10.3390/foods8120691

**Published:** 2019-12-17

**Authors:** Lira Souza Gonzaga, Dimitra L. Capone, Susan E.P. Bastian, Lukas Danner, David W. Jeffery

**Affiliations:** 1 Department of Wine and Food Science, The University of Adelaide (UA), PMB 1, Glen Osmond, South Australia 5064, Australia; lira.souzagonzaga@adelaide.edu.au (L.S.G.); dimitra.capone@adelaide.edu.au (D.L.C.); lukas.danner@adelaide.edu.au (L.D.); 2 Australian Research Council Training Centre for Innovative Wine Production, UA, PMB 1, Glen Osmond, SA 5064, Australia

**Keywords:** sensory assessment, web scraping, wine expert, wine review, wine score, text mining

## Abstract

Understanding the sensory attributes that explain the typicity of Australian Cabernet Sauvignon wines is essential for increasing value and growth of Australia’s reputation as a fine wine producer. Content analysis of 2598 web-based wine reviews from well-known wine writers, including tasting notes and scores, was used to gather information about the regional profiles of Australian Cabernet Sauvignon wines and to create selection criteria for further wine studies. In addition, a wine expert panel evaluated 84 commercial Cabernet Sauvignon wines from Coonawarra, Margaret River, Yarra Valley and Bordeaux, using freely chosen descriptions and overall quality scores. Using content analysis software, a sensory lexicon of descriptor categories was built and frequencies of each category for each region were computed. Distinction between the sensory profiles of the regions was achieved by correspondence analysis (CA) using online review and expert panellist data. Wine quality scores obtained from reviews and experts were converted into Australian wine show medal categories. CA of assigned medal and descriptor frequencies revealed the sensory attributes that appeared to drive medal-winning wines. Multiple factor analysis of frequencies from the reviews and expert panellists indicated agreement about descriptors that were associated with wines of low and high quality, with greater alignment at the lower end of the wine quality assessment scale.

## 1. Introduction

Typicity is a term that indicates the degree to which the characteristics of a wine reflect its delimited geographical area, and is influenced by terroir, grape variety, and viticultural and winemaking techniques [1]. It can be used to define a specific class of wine and embody the individuality of a wine profile [2], enabling wines from distinctive regions to be differentiated, identified and recognised [1]. Trading wine on the basis of typicity has become important for “Old-World” European wine producing countries such as France, where provenance and wine regional typicity are valuable tools used to recognise high quality wines [3]. Wine regions in Europe are also synonymous with certain grape varietals, but it is “New-World” producers that have tended to use varietals as a means of differentiating their wines [4]. This has been the case in Australia, a successful New-World country that occupied fifth place for global production (accounting for 5%) in 2016 [5]. However, that is not to say that provenance is an unrecognised concept in places like Australia, where wine producing regions and their wines are protected and represented by their geographical indication (GI) as defined by the Wine Australia Act 2013 [6].

Notably, wines possess a high level of aroma and flavour complexity due to the large compositional influences of grape origin, in combination with climate, differences among cultivars and cultural practices. Due to its complexity, wine is available in a wide array of styles and prices from numerous brands, which also attests to the willingness of consumers to look for and pay for products that are tailored to suit their expectations [7]. As alluded to for Old-World wines, consumers use place of origin information as a cue for quality, and it also influences their willingness to pay [8,9]. However, the indication of place of origin is no less important for New-World wine consumers [10] independent of their involvement with wine [11]. These findings are also tied in with the approach increasingly emphasised by the Australian wine industry and supported by the Wine Australia strategic plan for 2015–2020, which is to promote regional branding and delimitation of regions so each local style and variety can be easily recognisable and distinguished [12].

In order to meet consumers’ wine flavour preferences, a better understanding of wine regionality and typicity from a sensory perspective is important. Wine professionals are often used for such studies but the pool of wines is generally small (i.e., up to two dozen wines but typically about half that number) and the criteria used to define the regions chosen for comparison is often not specified [3]. Although unexplored, it appeared that content analysis of wine writers’ descriptions could serve as a useful, rapid and cost-effective methodology to help screen and select representative regional wines for sensory assessment and determine regions to be benchmarked. Content analysis has been studied for decades and has proven useful in multiple fields, including political science [13], linguistics [14], health care [15,16], and consumer research [17]. Content analysis can be used in conjunction with text mining, a methodology that enables the extraction of information that has a context meaning from data that are textual and unstructured, and has been used to analyse social media content [18], consumers’ opinions on controversial topics [19], and smartphone applications [20]. Even though it is still not widely used in the wine and food science field, this methodology has been applied to understand consumer responses to mouthfeel attributes of wine [21], indicating that it could be useful more broadly for evaluating wine sensory characteristics. 

This research aimed to demonstrate how content analysis can be used as a tool to investigate regionally distinctive wine sensory profiles and to select regional wines for future studies, using the abundant tasting data that is available in online wine reviews. It also aimed to relate that data with the content analysis of freely written descriptors obtained from 11 wine professionals, who evaluated the sensory attributes of a set of 84 Cabernet Sauvignon wines from different regions of Australia and from Bordeaux. Overall quality judgements of the assessed wines were also used in the comparison.

## 2. Materials and Methods 

The regions were selected based on the relevance of the Cabernet Sauvignon variety for that GI, regional climate similarities (for the Australian regions), and benchmark indications provided by industry stakeholders. Consequently, the regions selected for this study were Coonawarra, Margaret River, and Yarra Valley, along with Bordeaux in the case of the expert assessment aspect of the study.

### 2.1. Wine Reviews

#### 2.1.1. Sample Selection

The selection of wine review sources was based on their relevance to the Australian wine market context, their online availability, and the existence of reviews of Cabernet Sauvignon wines from the Margaret River, Coonawarra, and Yarra Valley GIs of Australia across multiple vintages. Ultimately, the sources included Huon Hooke [22], Wine Spectator [23], Wine Enthusiast Magazine [24], Jeremy Oliver [25], James Halliday [26], and The Wine Front [27]. Bordeaux wines were included in the next phase of this study, but were not included in the first analysis due to the lack of chosen online sources that consistently reviewed wines from that region.

#### 2.1.2. Sample Extraction

A total of 8454 reviews were extracted using Octoparse web scraping software (version 7.1.2, Octopus Data Inc., Walnut, CA, USA). For each source of reviews, a unique workflow chart was created in the software to extract the required information. The data gathered by the software were downloaded into Microsoft Excel 2016 and input into Wordstat (version 8, Provalis Research, Montreal, QC, Canada). The software was programmed to extract the tasting notes and overall quality scores on a 100-point scale. To cope with the different numbers of reviews per region for each writer, a subset of reviews was randomly selected based on the lowest number of reviews per region for a writer, yielding 2598 reviews in total. For example, if a writer had written 460 reviews for Yarra Valley and more for each of the other regions, then 460 reviews for each of those other regions were randomly selected from the respective pool to derive the set of reviews for that writer. The chosen reviews were also restricted to vintages from 2001 through to 2016. 

### 2.2. Expert Panel

#### 2.2.1. Sample Selection 

A total of 84 commercially-available Cabernet Sauvignon wines were selected from the chosen regions based on the following criteria: vintage 2015; maximum retail price of AU$150; Cabernet Sauvignon in the blend, with a minimum of 60% for the Bordeaux and 85% for the Australian wines, respectively. Ultimately, the sample set consisted of 34 wines from Coonawarra, 20 from Margaret River, 20 from Yarra Valley, and 10 from Bordeaux. Within these criteria, the analysis of the online wine reviews served as a guide to identify samples that had a positive review, in order to avoid faulty wines, and that matched with the regionality profile found in the first part of the study.

#### 2.2.2. Sensory Analysis

An 11-member panel was composed of senior winemakers and/or experienced wine show judges and educators from Australia who were highly familiar with Cabernet Sauvignon wines and accustomed to tasting large sets of samples in one sitting. Sensory evaluation was conducted during a single day in an open plan focus group room designed for sensory analysis. Each wine sample (20 mL) was served in a clear XL5 wine tasting glass (Bormioli Luigi Glassmaker, Parma, Italy) randomly coded with a three-digit number and covered by a petri dish. The samples were randomised without considering region and divided into two flights, with 40 wines in the first and 44 wines in the second, and presented in a random order for each panellist. The first flight of wines was tasted between 9 am and 10:30 am. A 20 min break with light refreshments was provided before tasting the second flight from 10:50 am to 12:10 pm. The panellists were not aware of the regions nor sample selection criteria, but received instructions about the number of samples and the two tasks they were going to perform. For the first task, sheets with the random wine codes and spaces to hand-write descriptions were provided. The panellists were instructed to taste the wine and write down tasting notes using their own words for each sample. The second task involved scoring each sample out of 20 points based on the overall perceived quality. All the data were collected on paper and transcribed to produce an electronic version.

### 2.3. Data Preparation

The electronic version of the tasting notes and the online reviews were loaded into the content analysis software program. The program ran an automatic code on each raw dataset to exclude words that are usually not relevant for an analysis, such as prepositions, verbs, adverbs, etc. Prior to building a sensory lexicon, words that were not applicable to the analysis as a sensory attribute and that were not automatically excluded by the program (e.g., “Cabernet”, “Vintage” and “Wine”) were manually added to the exclusion list and were not considered for the analysis. 

A sensory lexicon was then created to agglomerate similar terms used by the wine writers and expert panellists into the same sensory category (Table 1) encompassing plurals and derivative words of the same attribute (e.g., blueberry and blueberries; dust and dusty) and keeping together words that represented the same category for the wine tasting context. Considering the expert panel data, only words used with a frequency of higher than five were considered in the categorisation. In the same way for the reviews, only words with a frequency higher than 50 were studied. All descriptors in the same category were analysed together, and each category was analysed based on its frequency within the region, to generate percentages. The data were exported to MS Excel for subsequent analysis with XLSTAT (Addinsoft, Paris, France, version 2019.1.1).

Overall quality scores were also prepared for analysis by aligning them with the Australian wine show judging criteria for awarding medals (Table 2) [28].

### 2.4. Statistical Analysis 

The same statistical analysis and lexicon categories were used to investigate tasting notes against regions and tasting notes against medals for the expert panel as well as for the wine reviews. Chi-square testing was performed with the word counts using Wordstat to determine categories that were significantly different between the regions as well as between medals. Before further evaluation, categories that had a count of < 5 for any region or assigned medal were disregarded. Assessing the relationship between the regions or medals and the descriptors, categories with *p*-values ≤ 0.1 were analysed through correspondence analysis (CA). A significance level of α = 0.1 was considered appropriate for the discrimination of categories due to the sensory nature of this study and the large set of samples evaluated by a small panel (in the case of the expert panel assessments) [29]. Multiple factor analysis (MFA) was conducted to compare the medal profiles using the significantly different categories (α = 0.1) that were common between the online reviews and expert panellists. MFA was performed in the same way with regional profiles to obtain regression vector (RV) coefficients and test how well the online review and expert panel datasets correlated. Chi-square tests were performed to examine the relationship between regions and medals along with Fisher’s exact test, considering α = 0.05. Apart from the chi-square tests performed with Wordstat, all statistical analyses were performed in XLSTAT. 

## 3. Results

The use of the web scraping software was essential in extracting a large number of online reviews in an adequate timeframe. A sensory lexicon was built in Wordstat by agglomerating words with the same meaning (in terms of a wine tasting lexicon) within the same category, yielding 47 categories (Table 1). With the categories assembled, Wordstat provided the percentages and chi-square results for each category (Tables S1 and S2 of the Supplementary Materials).

### 3.1. Regional Profiles of Cabernet Sauvignon Wines

**Figure 1:**
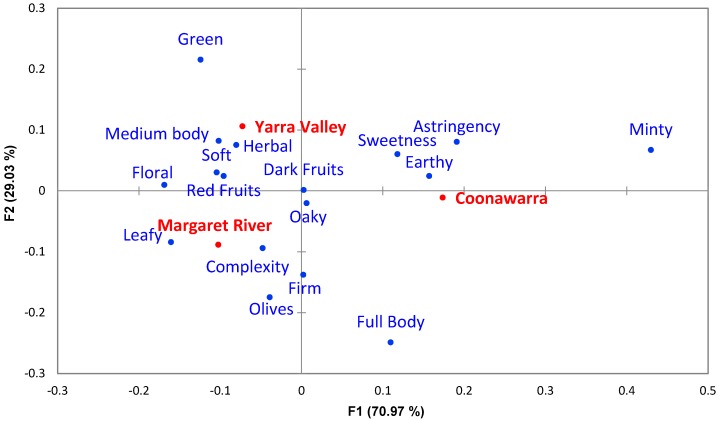
Correspondence analysis biplot for the online reviews of Cabernet Sauvignon wines based on significantly different descriptor categories (α = 0.1, chi-square test) for the three Australian regions.

Only online reviews for Australian wines underwent CA (Figure 1), which incorporated 17 significantly different categories (α = 0.1 and count ≥ 5; Table S1 of the Supplementary Materials). Axis F1 explained 70.97% of the data variance, and was mainly influenced by “Minty”, “Sweetness”, “Earthy”, “Full Body” and “Astringency” descriptors to the right and by “Floral”, “Soft”, “Red Fruits”, “Green”, “Herbal”, and “Medium Body” descriptors to the left. Axis F2 contributed 29.03% to the explained variance, mainly due to “Green”, “Medium Body”, “Herbal”, “Minty”, and “Astringency” in the top half of the biplot, in contrast to “Full Body”, “Olives”, “Firm”, “Complexity”, and “Leafy” in the bottom half. Some descriptors, such as “Dark Fruits” and “Oaky”, were not major drivers of the variance for these wines, as they were found close to the origin of the plot. With regard to the regions, Figure 1 shows Coonawarra loaded on the right side of the F1 axis, with Yarra Valley and Margaret River appearing opposite along F1. Axis F2 separated Margaret River in the bottom half of the plot from Yarra Valley in the top half (Figure 1). 

**Figure 2:**
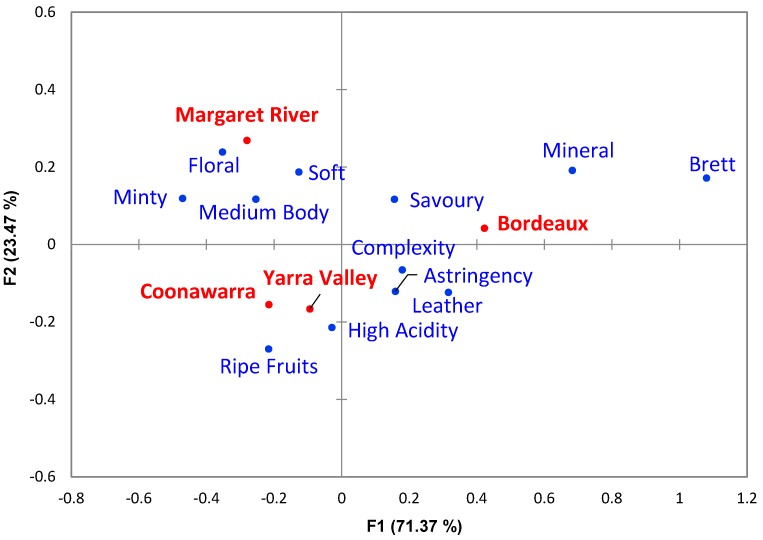
Correspondence analysis biplot for the expert panel assessment of Cabernet Sauvignon wines based on significantly different descriptor categories (α = 0.1, chi-square test) showing all four regions in this study.

CA was also performed using the frequency of 12 significantly different descriptor categories (α = 0.1 and count ≥ 5) from the expert panel tasting notes (Table S2 of the Supplementary Materials), using four wine regions that produce Cabernet Sauvignon (Figure 2). Axis F1 explained 71.37% of the variance found in the categories describing the wines, and was related to the descriptors “Brett”, “Mineral”, “Leather”, “Astringency”, “Savoury” and “Complexity” on the right along axis F1, as opposed to “Minty”, “Floral”, “Medium Body”, “Soft” and “Ripe Fruits” to the left. Axis F2 explained 23.47% of the variance found for the wines, and primarily related to “Floral”, “Soft”, “Mineral”, “Brett”, “Minty”, “Medium Body” and “Savoury” on the top of the plot. The lower part of axis F2 revealed the importance of “Ripe Fruits”, “High Acidity”, “Leather”, and “Astringency”. In terms of the regions, Bordeaux was on the right side of the plot along axis F1 whereas the three Australians regions appeared on the left. Furthermore, F2 shows separation of Margaret River along the top of the axis in comparison to Yarra Valley and Coonawarra, which can be found in the bottom half of the plot (Figure 2).

MFA was conducted with the significant descriptor categories (α = 0.1 and count ≥ 5) for the three Australian regions, using eight categories that were common between the two datasets (Figure S1 of the Supplementary Materials). This analysis yielded a moderate RV coefficient of 0.440, with F1 explaining 64.02% of the variation in the data and F2 contributing 35.98%. The projected mid-points for the regions (Figure S1a) showed that Coonawarra (having the best agreement between expert panel and online reviews) and Yarra Valley were separated from Margaret River along axis F1, whereas F2 separated Yarra Valley from the other two regions. Based on proximity of the descriptors, agreement was mostly evident for “Astringency” in the top right quadrant and “Medium Body” in the bottom left quadrant (Figure S1b), with other categories being located further apart but within the same half of the plot (along F1 or F2).

### 3.2. Medal Profiles

A chi-square significance test was performed on the wine reviews for assigned medals (based on a 100-point scale) against region in order to explore the relationship between quality (using medal as a proxy) and wine origin. With a *p*-value < 0.0001, the interaction between the regions and the frequencies of medals awarded was highly significant. Evaluating this further, Fisher’s exact test was performed on the data, based on observed frequencies, and theoretical frequencies for which no effect between medals and regions existed (Table 3). Results for Coonawarra were not significant, irrespective of the medal being considered. In comparison, Margaret River had a higher frequency of Gold medal wines and lower frequency of Bronze/No Medal wines, whereas Yarra Valley had a lower frequency of Gold medal wines and higher frequency of wines with no medals.

A chi-square test of significance was also performed on data from the expert panellists to assess the relationship between regions (including Bordeaux) and the frequencies of assigned medals based on a 20-point scale. In contrast to the online reviews, no significant difference was found in this case (*p*-value = 0.489).

**Figure 3:**
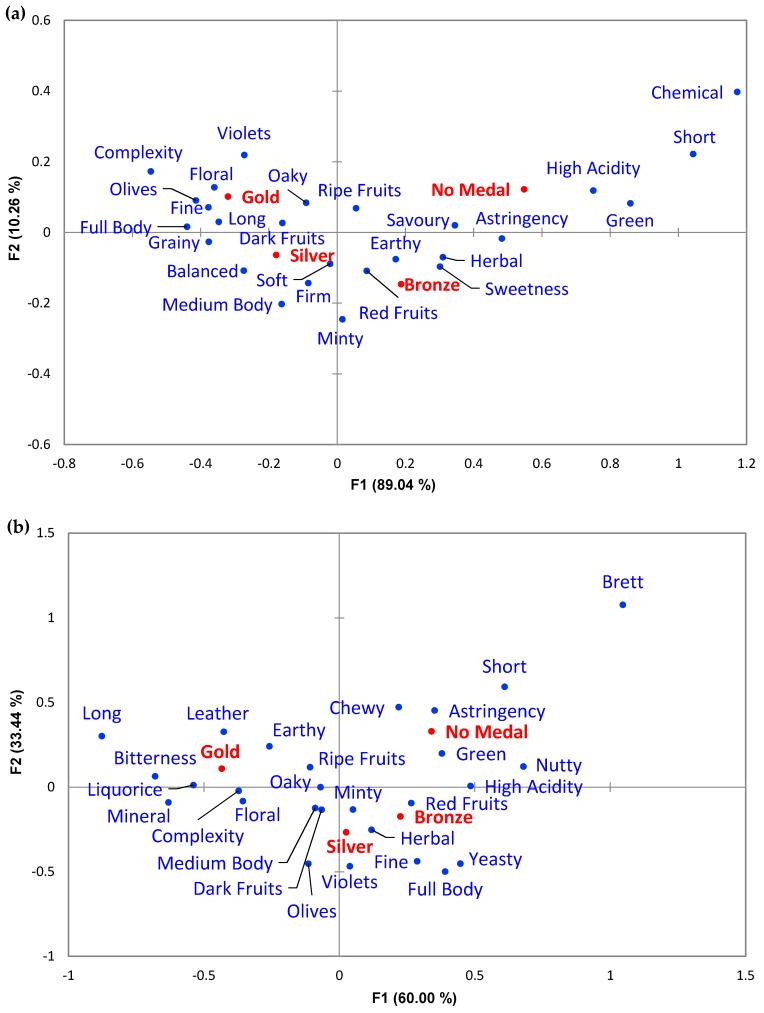
Correspondence analysis biplots of Cabernet Sauvignon wines from three Australian regions with significantly different descriptor categories based on the medals assigned (α = 0.1, chi-square test) for (**a**) the online reviews, and (**b**) the expert panel.

CA was performed according to assigned medal using the significantly different (α = 0.1 and count ≥ 5) descriptor categories, which totalled 26 attributes for the online reviews (Figure 3a) and 27 for the expert panel (Figure 3b). Considering the online reviews, F1 explained 89.04% of the variance of the data (Figure 3a), and was mainly related with “Chemical”, “Short”, “Green”, “High Acidity”, “Astringency”, “Savoury”, “Herbal”, “Sweetness, and “Earthy” attributes on the right side of the F1 axis, as opposed to “Complexity”, “Full Body”, “Olives”, “Fine”, “Grainy”, “Floral”, and “Long” on the left. This separated Silver and Gold medal wines to the left from Bronze and No Medal wines to the right. Axis F2 explained a further 10.26% of the sample variance (Figure 3a), mostly with respect to “Chemical”, “Violets”, “Short”, “Complexity”, “Floral”, and Long” attributes in the top half of the plot and “Minty”, “Medium Body”, “Firm”, “Balanced”, and “Red Fruits” below. Axis F2 distinguished Bronze from No Medal wines and Silver from Gold medal wines.

For the expert panel assessment, axis F1 explained 60.00% of the variance between the wines (Figure 3b) and was mostly defined by higher frequencies for “Brett”, “Nutty”, “Short”, “High Acidity”, “Astringency”, “Green”. “Yeasty”, “Red Fruits”, “Full Body”, and “Fine” on the right side. The opposite direction along F1 was mostly related to “Long”, “Bitterness”, “Mineral”, “Liquorice”, “Leather”, “Earthy”, and “Green” descriptors. Axis F1 was mainly able to differentiate Gold medals on the left, from Bronze and No Medal wines on the right. Axis F2 explained 33.44% of the variance between the wines (Figure 3b) and mainly related in the top half to higher frequencies for “Brett”, “Short”, “Chewy”, “Astringency”, “Long”, “Leather”, “Earthy”, and “Green” descriptors. On the opposite side along F2 were descriptors involving “Olives”, “Violets”, “Full Body”, “Fine”, and “Yeasty”. Attributes that were not cited were mostly close to the origin and did not have a large contribution to explaining the variance between samples. Axis F2 was mainly able to separate Bronze and Silver medal wines on the bottom half from No Medal wines on the top half of the plot.

**Figure 4:**
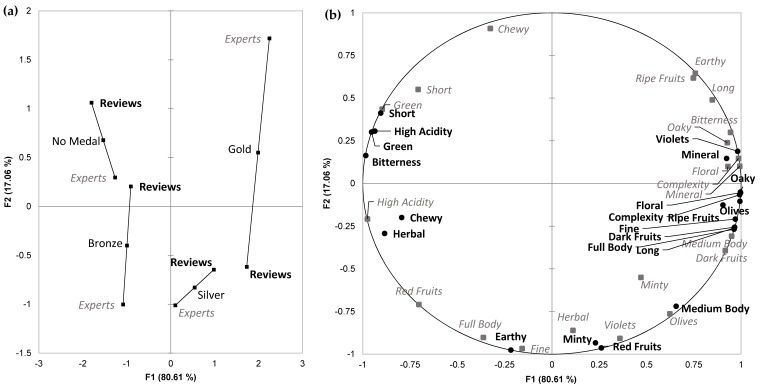
Multiple factor analysis plots of Cabernet Sauvignon wines with significantly different (α = 0.1, chi-square test) descriptor categories that were common between online reviews and expert panel showing (**a**) projected points of the assigned medals positioned about the mid-point in relation to the two descriptor datasets, where the length of the line is inversely related to the strength of the agreement, and (**b**) descriptors arising from the online reviews (●) and the expert panel assessments (■).

As with the regions, MFA was performed based on assigned medals and significantly different categories (α = 0.1) that were common to the expert assessments and the online reviews, to assess the agreement between the data from both cohorts (Figure 4). The projected points of the medals (Figure 4a) positioned Gold and Silver medals in the right quadrants of axis F1 (which explained 80.61% of the data variance) and Bronze and No Medal wines to the left. Axis F2 (17.06% explained) separated Gold and No Medal wines above the origin and Bronze and Silver medal wines below. Figure 4a also showed a good level of agreement between the wine reviews and the expert panel when assigning the medals (RV coefficient of 0.826), with the best agreement for Silver medals and the worst with Gold. Descriptors that were loaded on the MFA plot in close proximity and thus in agreement between the expert panel and the wine reviews were “Oaky”, “Mineral”, “Complexity”, “Floral”, “Dark Fruits”, “Green”, and “Short” (Figure 4b).

## 4. Discussion

This study sought to evaluate whether content analysis based on wine sensory attributes could differentiate wines from different regions and generate a guide for sample selection of regional wines for this and future studies. This was undertaken for Cabernet Sauvignon wines from selected Australian regions by assessing descriptors from online wine reviews in comparison to those arising from sensory assessment of wines by an expert panel. The approach was also utilised to examine sensory descriptors based on assigned medal (derived from 20- and 100-point scoring scales) for deeper insights into how overall Cabernet Sauvignon wine quality was perceived. Bordeaux red wines were included in the expert panel assessment for comparison and benchmarking with the Australian wines because Bordeaux is a renowned Old-World wine region with a strong appellation system and substantial production of Cabernet Sauvignon wines of quality. Bordeaux red wines are blends of red grape varietals, so for practical reasons, a minimum of 60% Cabernet Sauvignon was set as a selection criterion.

### 4.1. Regional Sensory Profiles for the Online Reviews and the Expert Panel

CA of the significant descriptors obtained from online reviews (Figure 1) revealed a distinction between the three Australian regions analysed in this study. Margaret River was characterised by “Leafy”, “Complexity” and “Floral” descriptors and less frequently described with “Minty” and “Astringency”. Coonawarra was described more frequently with the terms “Minty”, “Astringency”, “Sweetness”, and “Earthy” and less described with “Soft”, “Red Fruits”, and “Floral”. Yarra Valley was related to “Herbal”, “Green”, and “Medium Body” and less frequently described as “Full body” and “Firm”. CA (Figure 1) showed that the descriptors “Oaky” and “Dark Fruits” were plotted in close proximity to the origin of the plot and at a similar distance between the samples, showing that comparable frequencies were found between the regions. This is understandable, especially when dealing with commercial wines, because winemakers tend to aim for the presence of “Dark Fruits” attributes that can be considered as a marker of quality, as noted by Hopfer and Heymann [30] and borne out with an assessment based on medals (see Section 4.2). “Oaky” descriptors are associated with a winemaker’s decision regarding oak contact (e.g., barrel maturation or use of chips or staves), generating notes of “Vanilla”, “Buttery”, and “Caramel” depending on the raw material origin and prior treatment of the wood (e.g., heating, toasting, seasoning) [31,32]. Oak treatment is commonly used to increase mouthfeel and aroma complexity, and help stabilise colour [33], with a desirable influence as indicated by Hopfer and Heymann [30], Crump, et al. [34] and reinforced in the present study (see Section 4.2). Mouthfeel attributes like “Astringency” and “Soft” noted above are usually associated with the presence and structure of phenolic compounds, especially tannins that are present in grape skins and seeds [35]. The “Green” and “Herbal” descriptors characterising the Yarra Valley region were deemed to be related to 3-isobutyl-2-methoxypyrazine (IBMP), a compound commonly found in Cabernet Sauvignon wines and noted for its low odour detection threshold [36]. IBMP in wine originates directly from the grapes, and its concentration is influenced largely by viticultural practices and environmental conditions [37,38,39]. The influence of climate can be inferred by considering that the Yarra Valley is characterised by a cooler mean temperature and lower heat summation compared to the other two Australian regions [40].

Conducting CA in the same way on the expert panel assessment, which included wines from Bordeaux (Figure 2), showed clear separation along F1 of the Australian regions from Bordeaux, which appeared on the opposite side of the plot. This suggested that the descriptors used by the experts for the Australian wines were more similar than those attributed to wines from Bordeaux. A possible explanation relates to the divergence of Old-World countries compared to New World countries with respect to winemaking practices (e.g., fermentation processes, maturation techniques, and blending likely being the most important of all due to Bordeaux wines being composed of red blends), environmental conditions (e.g., soil, climate, and topography), and viticulture practices (e.g., pruning regime, irrigation, and training system) [41]. From Figure 2 it is possible to imply that Bordeaux wines were more frequently related to characters such as “Brett”, “Mineral”, and “Savoury”, whereas Coonawarra and Yarra Valley were related to “Ripe Fruits” and “High Acidity” descriptors. Coonawarra was less often described with the characters found in the Bordeaux wines in comparison to Yarra Valley, which was the closest region to Bordeaux in the plot and shared some attributes like “Leather”, “Astringency”, and “Complexity”. On the other hand, Margaret River was frequently associated with “Floral”, “Minty”, “Medium Body”, and “Soft”, but less so with “Astringency”, “Complexity”, and “Leather”. “Brett” and “Leather” (or less-frequently cited “Barnyard”, data not shown) terms used for the Bordeaux wines are often associated together [42] and originate from bioconversion of hydroxycinnamic acids present in the grapes [43], generating volatile phenols including 4-ethylphenol, 4-ethylguaiacol, and 4-ethylcathecol [44]. The compound 4-ethylcathecol has also been described as presenting a “Savoury” aroma [45]. The distinction of Australian GIs based on sensory attributes has been revealed before by Robinson, et al. [46], with regionality deemed in that work to have similarly influenced the sensorial properties of Cabernet Sauvignon wines. Although not apparent from the expert panel data of the present study, the work of Robinson, et al. [46] accords well with the “Minty” description cited in the wine reviews for Coonawarra Cabernet Sauvignon wines. Wine reviews and the expert panellists were aligned in their description of Margaret River wines as “Floral”, which is not clearly related to a single chemical compound in Cabernet Sauvignon wines, but could be influenced by monoterpenoids, C_13_-norisoprenoids, and sesquiterpenoids [47].

Evidently, the methods used to build the wine profiles were not identical and they were different sets of wine samples, making a direct comparison difficult. The expert panellists experienced a controlled environment where they evaluated the same bottle under the same blind conditions. With the wine reviews, descriptors were collated with no information on the conditions in which the wines were tasted, and the information was diffused through public media, which could have influenced the vocabulary and the descriptors used. The wine reviews would also portray vintage-to-vintage variation that influenced the overall profile of the regions, as opposed to the expert panel assessment that evaluated a snapshot from vintage 2015. Vintage variation of Cabernet Sauvignon wine sensory profiles has been demonstrated by Forde, et al. [48]. Considering the difference between both methods, it may be expected that not much concordance exists among the frequency of terms used, as was evident from the MFA results (Figure S1 of the Supplementary Materials) with only eight attributes in common for both datasets and a moderate RV coefficient of 0.440. Nonetheless, it was possible to see that both approaches could distinguish among the wine regions using the descriptors as drivers.

### 4.2. Quality Judgements for the Online Reviews and the Expert Panel 

Chi-square testing of the results from wine reviews showed that there was a significant interaction between the number of wines that were assigned a certain medal and the region of origin (Table 3). That interaction was evident for Margaret River and Yarra Valley wines, but not those from Coonawarra. A higher proportion of Margaret River wines were deemed to be Gold medal standard, which was contrasted by a lower proportion of wines that were assigned Bronze or No Medal. A somewhat opposite trend was revealed for Yarra Valley, whereby there was a lower proportion of wines assigned Gold medal and a higher proportion of wines with No Medal. This makes sense when considering the descriptors from the wine reviews associated with each region (i.e., Margaret River wines associated with positive traits like “Floral” and “Soft” terms in contrast to Yarra Valley wines being associated with “Herbal” and “Green” descriptors). It is not possible to ascribe this result to a true reflection of the quality expressed by the regions because it is unknown how the wines were evaluated, when reviews were written and how the wines were chosen to be evaluated by the reviewers. It could be possible that the reviews reflected some pre-conceived notion of the potential scores (and hence assigned medals) that each region should receive, if the evaluations were not conducted blind. The outcome from the online reviews contrasts with the results from the expert panel assessment, where the chi-square analysis showed no interaction between the medal counts and the regions. This implied that the overall quality scores from the expert panel were not strongly impacted by the regions where the wines arose. Even though the quality judgement presented in this study differs in methodology when compared to wine shows, it could be considered in line with the work of Allen and Germov [49], who demonstrated that judging of wine quality at Australian capital city wine shows appeared to be based on either national or international standards and not regional specifications.

CA of the assigned medals and the sensory descriptors from the wine reviews (Figure 3a and Table S1 of the Supplementary Materials) showed that Gold medal wines were associated with “Fine”, “Floral”, “Complexity”, “Long”, “Olives”, “Full Body”, “Violets” and “Oaky” descriptors, whereas Silver medal wines were more frequently associated with “Grainy” and “Balanced” and less so with “Green”, “Short”, and “Chemical”. The “Dark Fruits” descriptor was plotted between Silver and Gold medals wines, which might indicate that this descriptor is more associated with higher wine quality. On the opposite side of Figure 3a, Bronze medal wines were associated with “Earthy” “Red Fruits”, “Herbal”, and “Sweetness” descriptors and a lack of “Oaky” and “Complexity”, whereas wines that received No medal were associated with “Green”, “Short” “High Acidity”, and “Chemical”, but generally lacking in fruit characters. It is noteworthy to observe that many descriptors were plotted between Silver and Bronze wines, such as “Medium Body”, “Minty”, “Red Fruits”, “Earthy”, and “Herbal”, which may indicate that these descriptors could have a positive or negative impact on the quality perception of the wine, depending on the balance of these and other sensory attributes.

Analysing the expert panel assessment in the same way (Figure 3b and Table S2 of the Supplementary Materials), Gold medal wines stood out more prominently from the rest, with terms including “Complexity”, “Bitterness”, “Earthy”, “Long”, and “Leather”. Silver medal wines were linked to “Herbal”, “Violets”, “Minty”, “Full Body”, and “Olives”. Some descriptors such as “Dark Fruits”, “Medium Body”, “Oaky” and “Floral” were plotted between Silver and Gold medal wines, which might demonstrate their influence on the sensory profiles of wines that express higher quality. Bronze medal wines were more frequently associated with “Red Fruits” and “Yeasty” descriptors and less with “Earthy”, “Leather”, and “Long”, and wines that were assigned as No medal were more correlated with “Green”, “Astringency”, “Short”, and “Brett” and less with “Dark Fruits” and “Medium Body”. Terms such as “High Acidity” and “Nutty” were associated with both Bronze and No medal wines, implying that those attributes were not perceived by the expert panellists as quality characteristics for these wines. Some of the present findings accorded with those of Lattey, Bramley and Francis [7], in which wines with higher quality ratings were associated with “Minty”, “Spicy”, “Woody” (e.g., “Oaky”), “Complexity” and “Persistence” (e.g., “Long”), and lower quality wines were associated with *Brett*-related flavours of “Barnyard”, “Band-Aid” (e.g., “Chemical”) and “Leather”. These sorts of traits are associated with *Brettanomyces* spp., indigenous yeast species that can be present during wine fermentation or ageing and produce aroma compounds that impact on the finished wine [50]. The effect can be perceived as negative when in higher quantities or positive when contributing to the developed characters of the wine, which might explain the term “Leather” being frequently described by the expert panel [50,51]. It is also noteworthy to recall that wines tasted by the expert panel were 2015 commercial wines, so developed characters such as “Leather” (a tertiary character that does not necessarily have to arise from *Brettanomyces*) were to be expected. In relation to “Green” being correlated with No medal wines, Hopfer, et al. [52] showed that “Fresh Green” (and “Soy Sauce”) aromas had a negative impact on the quality ratings of wines. Furthermore, the attributes related to low quality ratings were also in alignment with the consumer preferences investigated by Ristic, et al. [53], whereby savoury-like flavours (e.g., “Gamey/ Meaty”, “Soy Sauce”, “Salami”, and “Barnyard”) and green-like flavours (e.g., “Capsicum”, “Green Beans”, “Green Peas”, “Green Tea”, and “Green Grass”) were the least favourite. Finally, Niimi, et al. [54] explained that higher perceived quality was linked to more complex, wines where sensory characteristics such as “Dark Fruit”, “Colour”, “Body” and “Astringency” were more intense. It was also implied by Niimi, et al. [54] that the balance of “Green” flavour drives quality ratings in Cabernet Sauvignon wines, where too much or too little can be perceived as negative. Overall, those observations align well with the present results.

### 4.3. Quality Judgment Correlation Between the Wine Reviews and Expert Panel

MFA was conducted to investigate the online review and expert panel descriptor datasets with respect to assigned medal (as a proxy for quality). It is interesting to note in Figure 4b that the expert panel and the wine reviewers agreed with their use of “Dark Fruits”, “Complexity”, “Oaky”, “Mineral” and “Floral” descriptors based on their close proximity, to the right along axis F1. As observed in Figure 4a, those attributes appeared to be important for wines that were perceived as higher in quality (i.e., assigned Gold or Silver medals). On the other hand, both sources of data agreed that “Green” and “Short” were attributes associated with wine perceived to be at the lower end of quality, and not receiving a medal. From Figure 4a it is clear that the expert panel could better differentiate the four medal classes, with each appearing in a distinct quadrant; this did not happen with the wine reviews. Nonetheless, strong agreement between both data sources was evident, with MFA producing an RV coefficient of 0.826. Agreement between the expert assessment and the online reviews was better when evaluating lower quality wines, with a particular contrast between wines assigned as No medal versus Gold medal (Figure 4a). This might imply that it is easier to judge (or agree upon) factors that have a negative impact on wine quality as opposed to traits that are indicators of higher quality wine. This assertion is supported by the work of Hodgson [55] and Allen and Germov [49], who evaluated wine competitions in Australia and USA, respectively. They concluded that wine judges had more concordance between wines that should receive a medal or not, as opposed to assigning Gold, Silver or Bronze medals to the wine. Overall, the results from MFA pointed to the potential validity of using content analysis as a new methodology to explore wine characterisation and wine typicity in an efficient and low-cost way, with a view to selecting wines for further study.

### 4.4. Concepts of Typicity in Relation to Quality

Quality is a multifaceted concept that involves a complex link between intrinsic (appearance, gustatory, origin, variety, typicity and potential) and extrinsic (grapes, production, marketing) characterisation [56]. This is corroborated by Verdú Jover, et al. [57], suggesting that red wine quality should be evaluated using a two-dimensional scale: one for extrinsic attributes (involving origin, image and presentation) and one for intrinsic attributes (involving age, harvest, sensitivity and acuteness). Typicity of a region could be a term that is connected directly with wine quality, although the scores were assigned and the respective tasting notes were obtained from the expert panel without any knowledge about the regions being evaluated. Even with the online reviews, it might be anticipated that there was limited prior knowledge of the wine being tasted. The present study therefore dealt only with some aspects of intrinsic characterisation, but still revealed attributes that might be able to profile sensory typicity, which features in one of the dimensions of quality. It was possible to connect some terms that were important both for assigned medals (proxy for quality) and for certain regions. For example, “Floral” that was a term highly cited for Gold medal wines and for Margaret River wines. However, further research is necessary to better understand the relationship between wine quality and typicity in a sensory manner.

## 5. Conclusions

The use of web scraping and content analysis software has been proposed as a valuable approach for investigating different sensory profiles of wines according to online sources in a rapid timeframe. The methodology can also serve as a selection guide of regional wines that can undergo more in-depth sensory work, such as by expert panellists. This aspect was demonstrated with commercial Cabernet Sauvignon wines from Margaret River, Coonawarra, Yarra Valley, and Bordeaux, which could be distinguished by different sensory profiles, indicating that terroir, viticulture and winemaking practices can have an impact on the final characteristics of the wine in a region-specific manner. 

Results from the medal assignments based on quality score showed that assessments by those with high involvement in the industry (i.e., wine reviewers or winemaker as in this study) can lead to a distinction between wines considered to be at the higher and the lower end of wine quality. Furthermore, based on the frequencies of the descriptors used, certain sensory characteristics appeared to be more important when judging the overall quality of the wine as “Complexity” and “Dark Fruits” for high quality and “Green” for lower quality.

MFA of frequencies obtained from the reviews and expert panellists revealed the extent of agreement between common descriptors from both data sources that were associated with the different regions and with wines of different ascribed quality. Based on RV coefficients, there was very good agreement for wines based on quality, with greater alignment at the lower end of the wine quality assessment scale, and moderate agreement based on region. The use of MFA helped to highlight the potential of using content analysis according to the proposed methodology to efficiently explore wine characterisation and typicity while minimising the costs of such an undertaking of the scale presented in this work.

Overall, this study has presented a strategy to select wines that will undergo further analysis, using sensory methods in this case, but the concept could be extended to exploring the chemical profiles of wines. Indeed, the approach will be exploited in upcoming trials to characterise the typicity of the Australian Cabernet Sauvignon wines from different origins. The present work lays the foundation that will become important to the wine industry when aiming to understand regionality and the associated unique attributes, which can serve as a tool to promote regional wines and attend to consumer demands for uniqueness. It also provides understanding of the existence of regional profiles and how these may relate to quality scores.

## Figures and Tables

**Table 1:** Categories and respective descriptors created in Wordstat to analyse content from wine reviews and expert sensory panellists (plurals and derivative words are not listed in this table but were considered in the analysis).

	Category	Descriptors	Category	Descriptors
Aroma/Flavour	Dark Fruits	Dark fruit, blackcurrant, blueberry, blue fruits, black cherry, black fruit, blackberry, cassis, juniper, mulberry, and plum.	Red Fruits	Red fruit, red cherry, red berry, red currant, boysenberry, cranberry, raspberry, and strawberry.
	Chemical	Band-Aid, boot polish, medicinal, metallic, ozone, plastic, petroleum, pungent, and soapy.	Herbal	Herbal, bay leaf, herbaceous, rosemary, tea, thyme, and sage.
	Ripe Fruits	Ripe berry, apricot, fruit cake, fig, jam, overripe, porty, prune, raisin, shrivelled, and ripe fruit.	Green	Green, dill, grass, capsicum, sappy, shaded, stalky, vegetal, and weedy.
	Savoury	Savoury, sea salt, barbeque sauce, beef stock, gamey, iodine, meat, oyster, pancetta, salty, salami, sea spray, seaweed, soy sauce, steak, and vegemite.	Oaky	Oaky, cigar box, coffee beans, burnt, butterscotch, caramel, cedar, chocolate, cocoa, coconut, mocha, tarry, vanilla, and woody.
	Nutty	Nuts, almond, chestnut, and nutty.	Peppery	Pepper, peppercorn, and white pepper.
	Smoky	Smoky, ashtray, charcoal, roasted and tobacco, guaiacol.	Confectionery	Confectionery, jelly, lolly, marshmallow, and juicy fruit.
	Floral	Floral, rose water, geranium, lavender, rose, Turkish delight, and perfumed.	Cooked Vegetables	Cooked vegetable, canned green bean, sulphide, vegetable, and eggplant.
	Minty	Minty and spearmint.	Sweetness	Sweet.
	Eucalyptus	Eucalyptus, gum leaf, camphor, and pine.	Leafy	Leafy, foliage, stem.
	Spicy	Spicy, clove, curry, cardamom, and ginger.	Citric	Jaffa, orange, chinotto, rhubarb, and zesty.
	Leather	Leather, barnyard, horse, and hay.	Violets	Violet and blue flower.
	Brett	Brett-related characters associated with *Brettanomyces* spp. activity.	Olives	Olive, tapenade.
	Earthy	Earthy, forest floor, dirt, dust, fungal, mossy, muddy, mushroom, musk.	Yeasty	Yeasty, barley, arrowroot, and toast.
	Mineral	Mineral, graphite, stone.	Apples	Apple and apple skin.
	Liquorice	Liquorice and anise.	Varietal	Varietal, typical, and Cabernet characters.
	Oxidative	Oxidised and aldehyde.		
Mouthfeel, Taste, Body	Soft	Soft, plush, silky, smooth, suede, and talc.	Firm	Firm and robust.
	High Acidity	High acidity, acidic, crisp, sour, and tart.	Grainy	Grainy, crunchy, powdery, and sandy.
	Fine	Fine tannins	Astringency	Astringent, drying, and puckering.
	Hotness	Hot and warming.	Balanced	Polished and rounded.
	Short	Short and fading.	Grippy	Grippy.
	Long	Lingering and persistent.	Chewy	Chewy.
	Complexity	Complex, layered, and structured.	Bitterness	Bitter, pips, and quinine.
	Medium Body	Medium body.	Full body	Full body, mouth filling.

**Table 2:** Scoring system used in online reviews and by expert panellists and their equivalence to wine medals based on the Australian wine show judging system [28].

100 Point Scale Used in Reviews	20 Point Scale Used by Expert Panellists	Medal Equivalent
95–100	18.5–20	Gold
<95–90	<18.5–17	Silver
<90–85	<17–15.5	Bronze
<85	<15.5	No medal

**Table 3:** Significance according to Fisher’s exact test from an assessment of assigned medal (derived from a score out of 100) in relation to region for the online wine reviews. Values in bold are significantly different from the theoretical frequency at the level α = 0.05. NS, not signifcant.

		Coonawarra		Margaret River		Yarra Valley	
Medal	Theoretical Frequency	Observed Frequency	Result	Observed Frequency	Result	Observed Frequency	Result
Gold	285	284	NS	**326**	**Higher**	**246**	**Lower**
Silver	409	404	NS	413	NS	410	NS
Bronze	131	143	NS	**103**	**Lower**	147	NS
No Medal	41	35	NS	**24**	**Lower**	**63**	**Higher**

## Supplementary Materials

The following are available online at https://www.mdpi.com/2304-8158/8/12/691/s1, Table S1: Frequencies of each categories for regions and medals based on the wine writers’ reviews along with the chi-square results and *p*-values; Table S2: Frequencies of each categories for regions and medals based on the expert panel assessment along with the chi-square results and *p*-values; Figure S1: Multiple factor analysis plot of Cabernet Sauvignon wines with common significantly different descriptor categories (α = 0.1, chi-square test) based on the regional profile.
