# Supplementary material for: Using Content Analysis to Characterise the Sensory Typicity and Quality Judgements of Australian Cabernet Sauvignon Wines
Source: Foods. 2019 Dec 17;8(12):691. doi: 10.3390/foods8120691 (PMC6963444; doi:10.3390/foods8120691)
Supplement: Supplementary file 1 [file foods-08-00691-s001.pdf]

SUPPLEMENTARY MATERIALS FOR

Using Content Analysis to Characterise the Sensory Typicity and Quality Judgements of Australian Cabernet Sauvignon Wines

Lira Souza Gonzaga [1,2], Dimitra L. Capone [1,2], Susan E. P. Bastian [1,2], Lukas Danner [1], David W. Jeffery [1,2,*]

[1] Department of Wine and Food Science, The University of Adelaide, PMB 1, Glen Osmond, South Australia 5064, Australia

[2] Australian Research Council Training Centre for Innovative Wine Production, The University of Adelaide, PMB 1, Glen Osmond, South Australia 5064, Australia

* Correspondence: david.jeffery@adelaide.edu.au

Table of Contents

Page

Table S1. Frequencies of each category for regions and medals based on the online wine writers' reviews along with the chi-square results and *p*-values. **S-2**

Table S2. Frequencies of each category for regions and medals based on the expert panel assessment along with the chi-square results and *p*-values. **S-4**

Figure S1. Multiple factor analysis plot of Cabernet Sauvignon wines with common significantly different descriptor categories based on the regional profile. **S-6**

Table S1. Frequencies of each category for regions and medals based on the online wine writers' reviews along with the chi-square results and *p*-values (significantly different categories with α = 0.1 with a count of 5 or more are shown in bold). Observed values that are higher than the theoretical value are shown in red and those that are lower are shown in blue.

Wine Reviews	Region					Medals					
Categories	Coonawarra	Margaret River	Yarra Valley	Chi-square	*p*-value	Gold	Silver	Bronze	No Medal	Chi-square	*p*-value
Dark Fruits	**72.40%**	**73.21%**	**68.48%**	**5.436**	**0.066**	**82.71%**	**74.57%**	**48.35%**	**33.61%**	**247.096**	**<0.001**
Chemical	1.50%	1.27%	1.04%	0.737	0.692	**0.47%**	**0.73%**	**2.80%**	**7.38%**	**50.823**	**<0.001**
Ripe Fruits	23.67%	21.71%	20.79%	2.190	0.335	**23.36%**	**23.15%**	**17.05%**	**18.03%**	**8.582**	**0.035**
Savoury	16.05%	18.01%	16.74%	1.220	0.543	**14.14%**	**17.52%**	**19.59%**	**22.13%**	**9.385**	**0.025**
Nutty	0.35%	0.58%	0.92%	2.390	0.303	0.47%	0.98%	0.00%	0.00%	6.129	0.105
Smoky	9.24%	9.01%	9.58%	0.174	0.917	9.46%	9.62%	6.87%	12.30%	4.229	0.238
Floral	**8.43%**	**12.70%**	**11.66%**	**8.832**	**0.012**	**14.84%**	**10.84%**	**5.09%**	**3.28%**	**34.533**	**<0.001**
Minty	**24.02%**	**8.78%**	**12.01%**	**87.937**	**<0.001**	**11.80%**	**17.36%**	**16.54%**	**7.38%**	**18.585**	**<0.001**
Eucalyptus	2.54%	3.12%	1.50%	4.990	0.082	1.75%	3.10%	1.78%	1.64%	5.047	0.168
Spicy	8.66%	8.31%	7.62%	0.644	0.725	8.76%	8.31%	7.63%	4.92%	2.293	0.514
Leather	1.96%	1.27%	0.58%	6.630	0.036	0.82%	1.63%	1.02%	1.64%	2.996	0.392
Brett	0.12%	0.23%	0.00%	2.002	0.367	0.12%	0.16%	0.00%	0.00%	0.836	0.841
Earthy	**25.17%**	**17.78%**	**18.48%**	**17.718**	**<0.001**	**17.17%**	**22.66%**	**21.63%**	**18.03%**	**10.087**	**0.018**
Mineral	3.58%	2.08%	2.19%	4.742	0.093	3.39%	2.69%	1.02%	1.64%	6.421	0.093
Liquorice	2.66%	1.50%	1.04%	7.056	0.029	1.75%	1.87%	1.02%	2.46%	1.705	0.636
Oxidative	0.00%	0.00%	0.46%	8.012	0.018	0.00%	0.08%	0.51%	0.82%	8.477	0.037
Red Fruits	**21.02%**	**26.10%**	**25.17%**	**6.937**	**0.031**	**20.68%**	**27.14%**	**24.17%**	**17.21%**	**14.844**	**0.002**
Herbal	**17.21%**	**19.63%**	**21.36%**	**4.830**	**0.089**	**16.00%**	**19.56%**	**25.45%**	**22.13%**	**16.099**	**0.001**
Green	**6.70%**	**7.39%**	**10.85%**	**11.270**	**0.004**	**3.74%**	**6.85%**	**17.56%**	**25.41%**	**117.804**	**<0.001**
Oaky	**66.28%**	**67.90%**	**60.28%**	**12.248**	**0.002**	**77.80%**	**64.79%**	**44.53%**	**39.34%**	**168.956**	**<0.001**
Peppery	3.00%	1.85%	1.15%	7.692	0.021	1.52%	2.12%	2.54%	2.46%	1.825	0.610
Confectionery	0.23%	0.58%	0.69%	2.010	0.366	0.12%	0.65%	0.51%	1.64%	6.275	0.099
Cooked Vegetables	0.23%	0.81%	1.39%	7.201	0.027	0.58%	0.65%	1.53%	1.64%	4.491	0.213
Sweetness	**12.24%**	**9.01%**	**10.16%**	**4.961**	**0.084**	**7.59%**	**11.57%**	**12.98%**	**11.48%**	**11.916**	**0.008**
Leafy	**10.05%**	**15.94%**	**11.89%**	**14.243**	**0.001**	12.27%	13.37%	12.21%	9.02%	2.211	0.530
Citric	0.46%	0.12%	0.46%	2.007	0.367	0.23%	0.33%	0.51%	0.82%	1.422	0.700
Violets	7.74%	9.82%	9.24%	2.452	0.294	**13.20%**	**7.91%**	**4.33%**	**4.10%**	**34.53**	**<0.001**

Table S1. contd.

Wine Reviews	Region					Medals					
Categories	Coonawarra	Margaret River	Yarra Valley	Chi-square	*p*-value	Gold	Silver	Bronze	No Medal	Chi-square	*p*-value
Olives	**7.62%**	**9.82%**	**5.89%**	**9.351**	**0.009**	**10.63%**	**7.66%**	**3.82%**	**1.64%**	**24.75**	**<0.001**
Yeasty	3.93%	4.16%	4.16%	0.079	0.961	5.37%	3.50%	3.31%	3.28%	5.499	0.139
Apples	0.35%	0.00%	0.00%	6.007	0.05	0.23%	0.08%	0.00%	0.00%	1.755	0.625
Varietal	8.78%	9.58%	10.16%	0.975	0.614	9.11%	10.02%	10.18%	4.92%	3.729	0.292
Soft	**18.59%**	**23.44%**	**22.86%**	**7.170**	**0.028**	**21.14%**	**23.47%**	**19.85%**	**12.30%**	**9.582**	**0.022**
High Acidity	2.54%	2.31%	3.70%	3.450	0.178	**1.52%**	**2.77%**	**4.58%**	**7.38%**	**18.797**	**<0.001**
Fine	20.90%	21.71%	24.36%	3.281	0.194	**27.69%**	**24.21%**	**9.92%**	**5.74%**	**70.905**	**<0.001**
Hotness	1.85%	0.46%	1.27%	7.117	0.028	0.93%	1.55%	0.76%	0.82%	2.56	0.465
Short	1.27%	0.92%	1.73%	2.205	0.332	**0.47%**	**0.90%**	**3.05%**	**5.74%**	**34.098**	**<0.001**
Long	5.77%	7.39%	5.89%	2.369	0.306	**7.71%**	**6.85%**	**3.31%**	**1.64%**	**13.837**	**0.003**
Complexity	**10.97%**	**13.51%**	**9.93%**	**5.784**	**0.055**	**16.24%**	**11.74%**	**3.31%**	**1.64%**	**56.645**	**<0.001**
Medium Body	**18.94%**	**22.63%**	**24.83%**	**8.903**	**0.012**	**21.14%**	**24.94%**	**20.36%**	**6.56%**	**23.984**	**<0.001**
Firm	**13.28%**	**15.24%**	**10.05%**	**10.644**	**0.005**	**11.80%**	**14.83%**	**11.20%**	**5.74%**	**11.618**	**0.009**
Grainy	12.47%	13.16%	12.01%	0.533	0.766	**14.37%**	**14.18%**	**6.62%**	**2.46%**	**29.488**	**<0.001**
Astringency	**9.82%**	**6.00%**	**7.39%**	**9.027**	**0.011**	**5.49%**	**7.74%**	**11.20%**	**12.30%**	**16.189**	**0.001**
Balanced	7.04%	7.27%	5.20%	3.696	0.158	**6.66%**	**7.58%**	**4.33%**	**1.64%**	**10.18**	**0.017**
Grippy	3.58%	2.66%	2.42%	2.307	0.316	0.93%	3.59%	4.33%	4.92%	18.474	<0.001
Chewy	2.66%	0.92%	0.81%	12.872	0.002	0.47%	2.04%	1.53%	2.46%	9.549	0.023
Bitterness	2.08%	1.50%	1.96%	0.891	0.640	0.58%	1.87%	3.56%	4.92%	20.255	<0.001
Full Body	**13.16%**	**13.39%**	**6.35%**	**28.400**	**<0.001**	**13.67%**	**11.82%**	**5.34%**	**1.64%**	**30.897**	**<0.001**

Table S2. Frequencies of each category for regions and medals based on the expert panel assessment along with the chi-square results and *p*-values (significantly different categories with α = 0.1 with a count of 5 or more are shown in bold). Observed values that are higher than the theoretical value are shown in red and those that are lower are shown in blue.

Expert Panel	Regions						Medals					
Categories	Bordeaux	Coonawarra	Margaret River	Yarra Valley	Chi-square	*p*-value	Gold	Silver	Bronze	No Medal	Chi-square	*p*-value
Dark Fruits	37.27%	39.49%	30.45%	36.82%	5.034	0.169	**49.06%**	**49.10%**	**34.87%**	**26.59%**	**36.745**	**<0.001**
Chemical	4.55%	6.58%	5.91%	8.64%	2.375	0.498	5.66%	7.53%	7.66%	5.74%	1.231	0.746
Ripe Fruits	**18.18%**	**30.13%**	**15.91%**	**27.27%**	**18.657**	**<0.001**	**41.51%**	**21.86%**	**23.37%**	**25.08%**	**9.557**	**0.023**
Savoury	**46.36%**	**18.73%**	**27.73%**	**25.00%**	**35.323**	**<0.001**	32.08%	28.67%	26.44%	21.45%	5.631	0.131
Nutty	5.45%	4.05%	5.00%	4.55%	0.537	0.911	**0.00%**	**5.02%**	**2.68%**	**6.65%**	**7.920**	**0.048**
Smoky	0.91%	1.01%	0.91%	1.36%	0.274	0.965	3.77%	0.36%	0.38%	1.81%	7.793	0.050
Floral	**5.45%**	**7.59%**	**11.82%**	**5.91%**	**6.765**	**0.080**	**18.87%**	**9.68%**	**9.20%**	**3.93%**	**17.52**	**0.001**
Minty	**6.36%**	**17.22%**	**19.09%**	**10.00%**	**15.340**	**0.002**	**16.98%**	**16.13%**	**18.39%**	**11.18%**	**6.571**	**0.087**
Eucalyptus	0.91%	1.52%	2.27%	1.82%	0.943	0.815	1.89%	1.08%	3.45%	0.91%	6.559	0.087
Spicy	11.82%	13.92%	10.00%	14.09%	2.415	0.491	9.43%	14.34%	12.26%	12.69%	1.189	0.756
Leather	**17.27%**	**6.58%**	**4.55%**	**10.45%**	**18.675**	**<0.001**	**24.53%**	**7.17%**	**4.21%**	**10.27%**	**25.799**	**<0.001**
Brett	**14.55%**	**0.76%**	**0.91%**	**1.36%**	**72.707**	**<0.001**	**0.00%**	**0.00%**	**1.15%**	**5.74%**	**25.871**	**<0.001**
Earthy	20.00%	19.24%	12.73%	20.00%	5.452	0.142	**39.62%**	**17.56%**	**10.73%**	**21.45%**	**28.436**	**<0.001**
Mineral	**8.18%**	**1.27%**	**1.82%**	**1.36%**	**20.555**	**<0.001**	**7.55%**	**3.94%**	**1.53%**	**0.60%**	**14.934**	**0.002**
Liquorice	0.91%	1.77%	0.91%	3.64%	5.319	0.150	**5.66%**	**3.23%**	**0.77%**	**1.21%**	**9.065**	**0.028**
Oxidative	2.73%	2.53%	3.64%	1.82%	1.446	0.695	1.89%	2.15%	2.30%	3.32%	1.107	0.775
Red Fruits	47.27%	47.59%	46.36%	44.09%	0.731	0.866	**30.19%**	**48.03%**	**49.43%**	**43.20%**	**8.041**	**0.045**
Herbal	9.09%	12.91%	14.09%	12.73%	1.692	0.639	**11.32%**	**14.70%**	**16.48%**	**7.85%**	**11.552**	**0.009**
Green	17.27%	18.23%	19.55%	15.00%	1.706	0.636	**11.32%**	**10.75%**	**18.39%**	**23.26%**	**18.015**	**<0.001**
Oaky	40.00%	39.24%	32.27%	43.64%	6.216	0.102	**58.49%**	**41.22%**	**39.85%**	**34.44%**	**11.908**	**0.008**
Peppery	2.73%	3.54%	2.27%	4.55%	1.914	0.591	0.00%	1.79%	4.60%	4.53%	6.367	0.095
Confectionery	5.45%	3.54%	5.45%	5.45%	1.890	0.596	0.00%	5.38%	3.83%	5.74%	4.079	0.253
Cooked Vegetables	4.55%	7.85%	6.82%	10.45%	4.056	0.255	1.89%	7.89%	7.66%	8.76%	3.015	0.389
Sweetness	9.09%	16.20%	12.27%	14.55%	4.369	0.224	15.09%	12.90%	14.18%	14.80%	0.510	0.917
Leafy	10.91%	12.41%	12.73%	10.91%	0.544	0.909	20.75%	11.11%	14.18%	10.27%	6.017	0.111
Citric	0.00%	2.78%	3.64%	2.27%	4.095	0.251	1.89%	3.94%	3.83%	0.60%	8.870	0.031
Violets	0.91%	5.57%	5.00%	4.09%	4.512	0.211	**3.77%**	**8.24%**	**4.60%**	**1.81%**	**14.219**	**0.003**

Table S2. contd.

Expert Panel	Regions						Medals					
Categories	Bordeaux	Coonawarra	Margaret River	Yarra Valley	Chi-square	*p*-value	Gold	Silver	Bronze	No Medal	Chi-square	*p*-value
Olives	0.00%	3.80%	2.73%	4.09%	4.849	0.183	**3.77%**	**6.09%**	**3.07%**	**0.91%**	**13.042**	**0.005**
Yeasty	6.36%	2.53%	3.18%	3.64%	3.933	0.269	**0.00%**	**6.45%**	**1.92%**	**2.72%**	**11.772**	**0.008**
Apples	3.64%	2.53%	2.73%	3.64%	0.819	0.845	0.00%	2.51%	4.60%	2.72%	4.206	0.240
Varietal	0.00%	3.80%	1.82%	2.27%	5.917	0.116	5.66%	2.51%	2.68%	2.11%	2.286	0.515
Soft	**24.55%**	**19.75%**	**28.18%**	**16.36%**	**10.589**	**0.014**	26.42%	20.79%	20.69%	21.75%	0.956	0.812
High Acidity	**19.09%**	**17.97%**	**10.45%**	**19.55%**	**8.354**	**0.039**	**5.66%**	**13.98%**	**18.01%**	**19.03%**	**7.797**	**0.050**
Fine	12.73%	9.87%	10.91%	6.36%	4.350	0.226	**3.77%**	**15.05%**	**10.73%**	**5.74%**	**17.237**	**0.001**
Hotness	5.45%	6.33%	5.91%	6.82%	0.288	0.962	9.43%	5.38%	7.28%	5.74%	1.891	0.595
Short	7.27%	5.06%	4.55%	4.09%	1.685	0.640	**1.89%**	**2.51%**	**2.68%**	**9.67%**	**22.478**	**<0.001**
Long	0.91%	1.77%	0.91%	0.91%	1.366	0.714	**7.55%**	**1.08%**	**0.77%**	**0.91%**	**17.227**	**0.001**
Complexity	**36.36%**	**22.03%**	**14.55%**	**18.18%**	**22.431**	**<0.001**	**49.06%**	**31.18%**	**14.18%**	**14.50%**	**57.381**	**<0.001**
Medium Body	**10.91%**	**10.63%**	**17.73%**	**15.00%**	**7.257**	**0.064**	**18.87%**	**17.92%**	**12.64%**	**9.67%**	**10.286**	**0.016**
Firm	9.09%	6.58%	4.09%	8.18%	4.201	0.241	5.66%	9.68%	5.75%	5.14%	5.715	0.126
Grainy	13.64%	12.66%	16.82%	12.27%	2.557	0.465	13.21%	14.34%	17.62%	10.57%	6.166	0.104
Astringency	**19.09%**	**12.15%**	**7.27%**	**11.36%**	**10.134**	**0.017**	**9.43%**	**6.09%**	**8.43%**	**19.94%**	**32.675**	**<0.001**
Balanced	1.82%	3.54%	3.64%	2.27%	1.586	0.663	1.89%	3.58%	3.45%	2.72%	0.729	0.866
Grippy	2.73%	3.04%	1.82%	4.09%	2.005	0.571	0.00%	1.79%	3.45%	3.93%	4.285	0.232
Chewy	9.09%	7.09%	7.73%	4.55%	2.985	0.394	**7.55%**	**3.58%**	**4.60%**	**11.78%**	**18.880**	**<0.001**
Bitterness	2.73%	3.54%	5.45%	3.18%	2.289	0.515	**15.09%**	**5.38%**	**2.68%**	**1.81%**	**24.248**	**<0.001**
Full Body	4.55%	7.34%	6.82%	8.18%	1.542	0.673	**1.89%**	**9.32%**	**10.73%**	**3.63%**	**15.203**	**0.002**

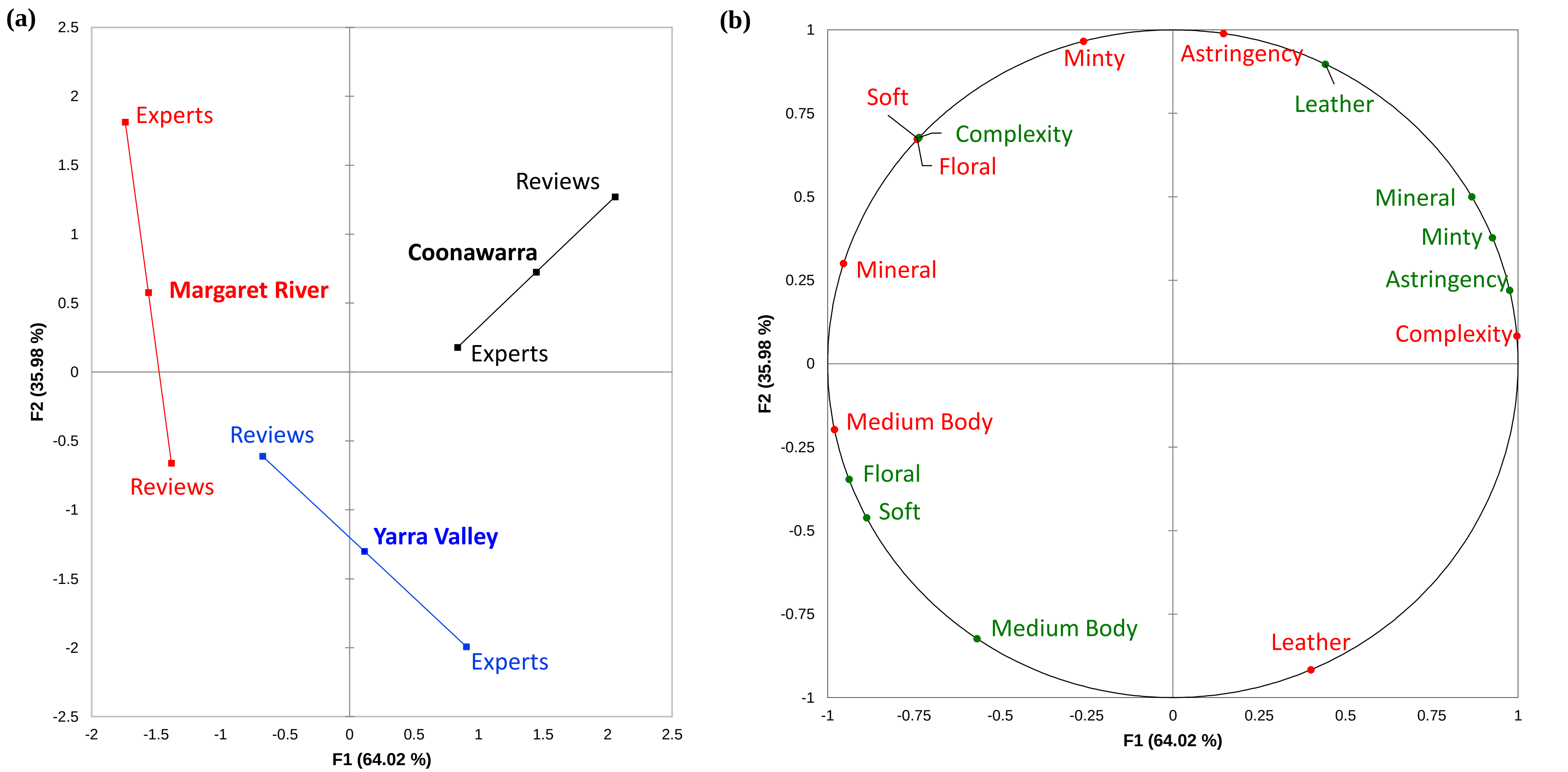

Figure S1. Multiple factor analysis plot of Cabernet Sauvignon wines with common significantly different descriptor categories (α = 0.1, chi-square test) based on the regional profiles showing (**a**) projected points of the regions according to online reviews and expert panel, where the length of the line is inversely related to the strength of the agreement, and (**b**) descriptors arising from the online reviews (green) and the expert panel assessments (red).
